# Supplementary material for: Silicone toothbrushes: A scoping review of an underutilized tool in global oral health
Source: PLOS Glob Public Health. 2026 Apr 22;6(4):e0005202. doi: 10.1371/journal.pgph.0005202 (PMC13102209; doi:10.1371/journal.pgph.0005202)
Supplement: Attachment — Submitted filename: Response to Reviewers.pdf [file pgph.0005202.s002.pdf]

Responses to Editor & Review Comments

Thank you for your insightful comments on our manuscript, PGPH-D-25-02407, entitled *"Silicone toothbrushes: A scoping review of an underutilized tool in global oral health."* We believe that incorporating the feedback from the academic editor and reviewers has substantially improved the manuscript. In particular, we have strengthened the discussion's narrative flow and enhanced methodological transparency, which together have improved clarity and reinforced the trustworthiness of our findings. Please find our detailed responses to each comment below.

Remove all section and subsection numbering throughout the manuscript (e.g., 3.2, 4.1, 4.2).

Response: We apologize about this error in the previous manuscript. All section and sub-section numbering have been removed.

Subheadings should appear only in the Methods and Results sections. All subheadings in the Discussion section must be removed.

Response: Subheadings were removed from the discussion section.

Structure the discussion as a continuous narrative guided by your key findings. Begin the Discussion section with a paragraph that presents the overall message of your findings. Each subsequent paragraph should: Start with a key message derived from your results, discuss similarities and differences with existing literature, and end with the public health and/or sustainability implications of the findings.

Response: We greatly appreciate this feedback and have dedicated considerable effort to restructuring the discussion section to improve narrative clarity and highlight the implications of our findings. This involved incorporating guiding sentences and reorganizing paragraphs to align more closely with the results section and create a more cohesive flow. In addition, following your suggestion, each paragraph now begins with a key message drawn from the results and concludes with its broader implications. For example:

"The findings this paper indicate the silicone toothbrushes are to traditional, nylon toothbrushes in plaque removal, which is a key component in maintaining oral hygiene (21,22,24,25,26,28,29)."

"Additionally, improper or inadequate toothbrushing is associated with increased risk of gingival trauma and tooth abrasion (21, 22). The results of the scoping review suggest that the soft and flexible bristle composition of silicone toothbrushes may mitigate these risks, representing a potentially more suitable oral hygiene strategy (21,22,26,28,29)."

"The oral health benefits of silicone toothbrushes, particularly for gingival health and plaque removal, warrant further investigation to better evaluate the effectiveness and broader applicability of silicone toothbrushes."

"As highlighted by 3 papers included in this scoping review, silicone toothbrushes have shown promise in addressing the unique oral health needs of underserved populations (21, 22, 26)."

Please revise this sentence in the limitation section for clarity: *"This may have led to omission of eligible literature that could have supported this paper's aim (i.e. (49))."* The reference to "(i.e. (49))" is unclear and likely erroneous. Consider rephrasing or removing the parenthetical citation.

Response: Thank you for catching this error. We have amended the sentence for clarity by adding additional details about the inserted citation: *"This may have led to omission of eligible literature that could have supported this paper's aim, for instance Kumar et al.'s paper exploring the effectiveness of a finger toothbrush (49)."*

In the Introduction section, the authors state that the study will present findings on the effectiveness, sustainability, and accessibility of silicone toothbrushes as an alternative to traditional approaches. However, the Results section does not clearly reflect findings related to accessibility. Please clarify the following:
- Were any findings presented that compare access to silicone toothbrushes versus traditional toothbrushes?
- Did the study identify any specific groups (e.g., by age, sex, location, or socioeconomic status) with greater or lesser access to silicone toothbrushes?
- If accessibility was not directly assessed or reported, consider revising the Introduction to reflect the actual scope of the findings. Alternatively, if relevant data exist, please incorporate and highlight accessibility-related results in the Results section.
This clarification is essential to ensure consistency between the stated objectives and the reported findings.

Response: This is an important point. We agree that the results section does not clearly reflect findings related to accessibility, as this aspect was not examined in the studies included in the scoping review. To maintain the manuscript's overall coherence, references to the accessibility of silicone toothbrushes have been removed from the introduction. Nevertheless, we acknowledge that accessibility is a critical dimension of this potential strategy and have clarified its relevance in the discussion, identifying it as a key gap in the existing literature to be addressed in future research.

Is Google Scholar an established database for grey literature?

Response: We agree that there are concerns regarding the use of Google Scholar as a database for grey literature. Accordingly, we searched five academic databases: PubMed, Web of Science, Scopus, CINAHL, and Embase. Google Scholar was not used as a formal database but rather as a supplementary tool for hand searching, given the novelty of the topic and the potential for relevant studies to be excluded from traditional indexing systems.

The search strategy used was for which database?

Response: Thank you for this question. In the manuscript, we clarify that *"the initial search strategy was built in EMBASE and then translated to the four other electronic databases"*.

Which authors did the data extraction and who solved the discrepancies?

Response: This is an important question, as it relates directly to the validity and rigor of the manuscript's results. Three authors were involved in data extraction, and all authors participated in resolving any discrepancies. Detailed author roles have been provided to *PLOS Global Public Health* during submission. To clarify which authors were involved in screening, we now state: *"Two independent reviewers conducted the abstract and full-text screening, with reviewer pairings varying across records."*

Sample size, outcome variables, and overall number of participants in each comparison, wherever applicable, is needed in the data extraction table.

Response: Thank you for this feedback. These are indeed crucial factors to consider, and they were extracted from the included articles wherever applicable. All extracted data is available at https://doi.org/10.5281/zenodo.16900885. We opted not to include this information in our summary of findings table to maintain overall clarity, given the wide variation in outcome variables and the inconsistent reporting of sample sizes and participant numbers across studies.

Possibility of meta-analysis can be assessed if outcome data is available

Response: Thank you for your suggestion. While meta-analyses are valuable for quantifying effects, the novelty of this topic and the limited, heterogeneous data currently available make a meta-analysis unfeasible. As such, we adopted a scoping review to systematically map and describe the available literature, rather than to synthesize effect sizes.

Image credits for figure 2 needed.

Response: We appreciate you catching this oversight. We now clarify that figure 2 was created by the authors. All icons used were royalty-free and sourced from Microsoft word.

While the introduction effectively highlights the global burden of oral diseases and the environmental concerns related to conventional toothbrushes, the emphasis on low- and middle-income countries (LMICs) as being disproportionately affected may need reconsideration. Oral diseases are indeed a global health issue, with high prevalence and significant impact across all income levels. The framing around LMICs might inadvertently narrow the perceived scope of the problem.

Response: Thank you for this insightful comment. We agree that oral diseases are a global health issue, affecting populations across all income levels. The emphasis on LMICs in our manuscript, however, was intentional and reflects the context in which silicone toothbrushes are being investigated as a potential strategy to reduce health inequities. In many LMICs, access to effective, affordable, and sustainable oral hygiene tools is limited due to economic, supply, and infrastructure constraints. By highlighting LMICs, we aim to underscore the potential public health impact of silicone toothbrushes in addressing these structural barriers, while not implying that oral health challenges are absent in higher-income settings. We have clarified this rationale in the introduction and discussion to ensure that the global relevance of oral disease is maintained, while also explaining why LMIC contexts are particularly important for evaluating the potential utility of this device.

The second paragraph of the Introduction currently reads as a list of materials rather than a cohesive narrative linking sustainability concerns to the specific potential of silicone. I recommend that the authors more clearly articulate why silicone, compared to other sustainable materials, may offer unique advantages for oral health applications.

Response: This is very helpful feedback. To enhance the clarity and coherence of the manuscript, in the introduction, we streamlined the discussion of materials and emphasized the distinctive properties of silicone relevant to oral health applications. These points are further elaborated in the discussion section (pp. 14–16). For example:

"Silicone's durability can extend a product's lifespan, thereby reducing the number of toothbrushes discarded in landfills. Silicone also offers greater potential for recycling and repurposing compared to conventional plastics (33)."

"As silicone has antimicrobial properties, this material could decrease the risk of contamination (17). Similarly to the silicone menstrual cup, a silicone toothbrush could be boiled for sterilization or cleaned with soap and water between uses (19)."

Additionally, the final sentences could better highlight the specific knowledge gap and research need that justify this scoping review.
Response: Thank you for raising this issue. We agree that we did not fully highlight the knowledge gap and research need. This has been added to the introduction:

"Peer-reviewed research on silicone toothbrushes is limited, generating a critical knowledge gap regarding their effectiveness, sustainability, and broader applicability in oral health. To address this gap, a comprehensive overview of existing literature is needed to evaluate the potential applications for silicone in the oral health domain. Accordingly, this paper summarizes the global literature published on silicone toothbrushes through a scoping review. Based on these findings, the potential of silicone toothbrushes as a viable solution for enhancing global oral health is explored."

Although a formal risk of bias assessment is not mandatory for scoping reviews, it is good practice to include a brief reflection on the methodological variability or potential limitations of the included studies.

Response: Great point. To support the trustworthiness and rigour of findings, we added more information regarding the methodological variability and potential limitations of included studies. For instance:

"*Fourth, four of the included articles were either conducted in laboratory settings, involved non-human participants, or were limited to life cycle assessment, restricting the ability of this review' to evaluate the real-world effectiveness and applicability of silicone toothbrushes."*

"Finally, the published literature on silicone toothbrushes is sparse and varies considerably in design, population characteristics, and outcome measures, which limits generalizability and the ability to draw firm conclusions. Overall, the heterogeneity and sparsity of literature highlight the need for more robust, comparative, and contextually grounded research."
